# c-Myc/microRNA-17-92 Axis Phase-Dependently Regulates PTEN and p21 Expression via ceRNA during Reprogramming to Mouse Pluripotent Stem Cells

**DOI:** 10.3390/biomedicines11061737

**Published:** 2023-06-16

**Authors:** Tomoaki Ishida, Tomoe Ueyama, Dai Ihara, Yukihiro Harada, Sae Nakagawa, Kaho Saito, Shu Nakao, Teruhisa Kawamura

**Affiliations:** 1 Department of Biomedical Sciences, College of Life Sciences, Ritsumeikan University, 1-1-1 Noji-higashi, Kusatsu 525-8577, Shiga, Japan; 2 Ritsumeikan Global Innovation Research Organization, Ritsumeikan University, 1-1-1 Noji-higashi, Kusatsu 525-8577, Shiga, Japan; 3 Department of Physiology, Tokai University School of Medicine, 143 Shimokasuya, Isehara 259-1193, Kanagawa, Japan

**Keywords:** induced pluripotent stem cells, microRNA, miR-17-92, c-Myc, PTEN, p21

## Abstract

Induced pluripotent stem cells (iPSCs) are promising cell sources for regenerative medicine and disease modeling. iPSCs are commonly established by introducing the defined reprogramming factors Oct4, Sox2, Klf4, and c-Myc. However, iPSC reprogramming efficiency remains low. Although recent studies have identified microRNAs that contribute to efficient reprogramming, the underlying molecular mechanisms are not completely understood. miR-17-92 is highly expressed in embryonic stem cells and may play an important role in regulating stem cell properties. Therefore, we examined the role of miR-17-92 in the induction of mouse iPSC production. c-Myc-mediated miR-17-92 upregulation increased reprogramming efficiency, whereas CRISPR/Cas9-based deletion of the miR-17-92 cluster decreased reprogramming efficiency. A combination of in silico and microarray analyses revealed that *Pten* and cyclin-dependent kinase inhibitor 1 (known as *p21*) are common target genes of miR-17 and miR-20a, which are transcribed from the miR-17-92 cluster. Moreover, miR-17-92 downregulated p21 in the early phase and PTEN in the mid-to-late phase of reprogramming. These downregulations were perturbed by introducing the 3′ UTR of PTEN and p21, respectively, suggesting that PTEN and p21 mRNAs are competing endogenous RNAs (ceRNA) against miR-17-92. Collectively, we propose that the c-Myc-mediated expression of miR-17-92 is involved in iPSC reprogramming through the phase-dependent inhibition of PTEN and p21 in a ceRNA manner, thus elucidating an underlying mechanism of iPSC reprogramming.

## 1. Introduction

Induced pluripotent stem cells (iPSCs) are generated by introducing reprogramming transcription factors such as, Oct4, Sox2, and Klf4 (OSK) or OSK with c-Myc (OSKM) [1,2]. Since iPSCs exhibit unlimited proliferation and pluripotency, they can be applied in disease modeling, drug development, and regenerative medicine [3]. However, their clinical applications are limited due to low production efficiency, clone–clone variability, and tumorigenicity. In particular, c-Myc, an oncogene and reprogramming factor, significantly increases the incidence of tumor formation and reprogramming efficiency. To address such issues, a deeper understanding of the mechanisms underlying iPSC production is necessary.

During reprogramming to iPSCs, OSKM induces dynamic changes in cellular characteristics and gene expression profiles [4]. Gene expression changes necessary for iPSC reprogramming are post-transcriptionally controlled by non-coding RNAs, including microRNAs (miRNAs) [5,6]. miRNAs are short (21–25 bases), single-stranded RNA molecules that play a crucial role in negatively regulating gene expression [7,8,9]. miRNAs bind to the complementary sequences of their target mRNAs, inhibiting further translation and triggering target mRNA degradation. miRNAs have diverse cellular functions, including proliferation and differentiation. Several miRNAs are also involved in efficient iPSC reprogramming [5,6,10,11,12]. The miR-290 cluster (miR-291-3p, miR-294, and miR-295) significantly increased iPSC production from mouse embryonic fibroblasts (MEFs) acting as downstream effectors of c-Myc [5]. miR-106b downregulated the expressions of p21 and TGFBR2 and enhanced reprogramming efficiency [6]. miR-302 and miR-372 contributed to iPSC production in human cells via the inhibition of TGFBR2 and RHOC [11]. Moreover, transient transfection with a combination of miR-200c, miR-302, and miR-367 also induced somatic cell reprogramming in mouse and human iPSCs without the use of OSKM expression vectors [12]. However, the involvement of miRNAs in iPSC production mechanisms is not fully understood.

miR-17-92 is a cluster composed of miR-17, miR-18a, miR-19a, miR-19b, miR-20a, and miR-92a, all of which are processed from the same precursor transcripts. miR-17-92 modulates the proliferation and survival of neural precursor cells by downregulating tumor suppressor genes [13]. miR-17-92 is also highly expressed in embryonic stem cells (ESCs) [14] and may play an important role in regulating stem cell properties. Therefore, in this study, we focused on the molecular mechanisms underlying the role of miR-17-92 in mouse iPSC production and identified the miRNAs transcribed from the miR-17-92 cluster that are most relevant to iPSC reprogramming. We show that c-Myc-mediated miR-17-92 upregulation enhances reprogramming efficiency through the phase-dependent downregulation of reprogramming suppressor genes in a post-transcriptional manner.

## 2. Materials and Methods

### 2.1. Plasmid Construction and Lentivirus-Mediated Genome Editing

Retroviral pMXs vectors (pMXs-mouse Oct4, pMXs-mouse Sox2, pMXs-mouse Klf4, and pMXs-mouse c-Myc) were used to produce mouse iPSCs from MEFs, as previously described [15,16]. For lentiviral vectors expressing miR-17-92 or individual miRNAs in the miR-17-92 cluster, we subcloned the loci from genomic DNA using a PCR-based method, as previously described [13]. Cloned DNA fragments were dissected with restriction enzymes, *Bam*HI and *Xho*I or *Nhe*I and *Bam*HI, and inserted into pLenti or pEGP vectors, respectively. pBabe-Puro-Cre, a retroviral vector encoding Cre recombinase, was also constructed by inserting the Cre cDNA into pBabe-Puro (addgene).

To construct luciferase reporter plasmids of PTEN 3′ UTR (full-length and region a, b, c, d, d, or f) or p21 3′ UTR (full-length), 3′ UTR sequences of each gene were amplified from mouse genomic DNA, and inserted into pcDNA3-Fluc MCS, which was subcloned by inserting Firefly luciferase cDNA into pcDNA3. pcDNA3-DsRed2-MCS was constructed by inserting a *Hind*III-DsRed2 cDNA-*Acc65*I fragment into pcDNA3. pcDNA3-DsRed2-PTEN 3′ UTR (full-length or region a, b, c, d, d, or f) and pcDNA3-DsRed2-p21 3′ UTR (full length) were prepared via the insertion of each 3′ UTR fragment into pcDNA3-DsRed2-MCS, respectively. To generate pLentiCAG-DsRed2-MCS, DsRed2 cDNA was inserted into pLentiCAG. pLentiCAG-DsRed2-PTEN 3′ UTR (full-length and region a, b, c, d, d, or f) was constructed by inserting each 3′ UTR fragment into pLentiCAG-DsRed2-MCS.

For genome editing with clustered regularly interspaced short palindromic repeat (CRISPR)/Cas9, lentiviral vectors were prepared using pLentiCRISPR (addgene), which expresses both Cas9 nuclease and short guide RNA (sgRNA) [17]. The CRISPR design tool (https://crispr.dbcls.jp/, accessed on 10 April 2022) was used to design sgRNA sequences to target the locus of miR-17-92. Oligonucleotides used for the sgRNA pair targeting the miR-17-92 cluster or c-Myc binding motif are available on request.

### 2.2. Cell Culture and Mouse iPSC Formation Experiments

MEFs were isolated from E13.5-E14.5 mouse embryos of C57BL/6 or *Pten*^fl/fl^ mice (Jackson Laboratory, Bar Harbor, ME, USA). MEFs were cultured in 10% FBS-containing medium supplemented with GlutaMAX (Gibco, Waltham, MA, USA) and penicillin–streptomycin solution (Wako, Osaka, Japan). Undifferentiated mouse ESCs (CGR8 cell line, purchased from Merck) and mouse iPSCs (established in a previous study [15]) were maintained on feeder cells in LIF-containing medium composed of DMEM (Nacalai, Kyoto, Lapan), 5% FBS (Molecular Probe, Eugene, OR, USA), 7.5% knockout serum replacement (Nichirei, Tokyo, Japan), GlutaMAX (Gibco, Waltham, MA, USA), penicillin–streptomycin solution (Wako), sodium pyruvate (Wako, Osaka, Japan), minimum essential non-essential amino acid (Gibco, Waltham, MA, USA), EmbryoMax nucleosides (Chemicon, Tokyo, Japan), and 0.2 mM 2-mercaptoethanol (Gibco, Waltham, MA, USA). Feeder cells were MEFs pretreated with mitomycin C (Sigma, Burlington, MA, USA) for 2 h.

For reprogramming to mouse iPSCs, retroviral vectors encoding Oct4, Sox2, Klf4, or c-Myc were prepared in packaging cells, HEK 293T cells (maintained in 10% FBS-containing medium supplemented with GlutaMAX (Gibco, Waltham, MA, USA) and 0.5 mg/mL geneticin 418) using Lipofectamine2000 Reagent (Thermo Scientific, Waltham, MA, USA). After 48 h of incubation, the retroviral vectors of the reprogramming factors were collected, and MEFs were treated with virus medium containing polybrene.

### 2.3. Quantitative Real-Time PCR

Total RNA samples were isolated from MEFs on day 5 of gene introduction, as were isolated from undifferentiated ESCs and iPSCs, using a Trizol-based method. cDNA was synthesized from up to 500 ng total RNA using a High-Capacity cDNA Reverse Transcription kit. To quantify the expression levels of miRNAs, a TaqMan microRNA Assay kit was used. To assess the expression level of other genes of interest, synthesized cDNA was amplified using SYBR^®^ Premix Ex Taq™ II (Tli RNaseH Plus). Primer pairs used for quantitative PCR are available on request.

### 2.4. Luciferase Reporter Assay 

For the luciferase assay, we used M50 super 8× TOP flash (addgene) as a template. The wild-type and mutated promoter regions of miR-17-92 were amplified via PCR and replaced with the pre-existing promoter sequence. DNA fragments of miR-17-92 promoter #1, miR-17-92 promoter #2, miR-17-92 promoter #3, or the wild-type/mutated promoter region of c-Myc were used. pSV40 *Renilla*-luc was used as a negative control. The indicated combination of the reporter plasmids or a single plasmid was transfected into MEFs or HEK 293T cells. Following 48 h of incubation, transfectants were subjected to a luciferase reporter assay using PicaGene Dual Sea Pansy Luminescence kit (TOYO B-NET, Tokyo, Japan) according to the manufacturer’s protocol. Raw luciferase values were normalized by the value of mock-transfected cells.

### 2.5. Chromatin Immunoprecipitation

Cultured cells infected with retroviral vectors prepared from pMXs-c-Myc-Flag ×3 were fixed in 1% formaldehyde for 10 min, and neutralized with 125 mM glycine. Following washing with D-PBS (-) twice, the cells were scraped and centrifuged. The cell pellet was subsequently processed in LB1 lysis buffer, LB2 wash buffer, and LB3 wash buffer containing a protease inhibitor cocktail too obtain the nuclear extract from the cultured cells. The samples were sonicated to fragment genomic DNA at 200–1000 bp and centrifuged. The supernatant was used as the input. The pellet was then incubated with anti-Flag M2-fused Protein G beads at 4 °C, overnight. Mouse normal IgG was used as a negative control. The samples were subsequently washed with low-salt buffer, an then, high-salt buffer, and solved in RIPA buffer, followed by elution and centrifugation. The supernatant was used as a ChIP sample. The ChIP samples were treated with RNase A and proteinase K. DNA in the samples was purified using a PCR purification kit. The enrichment of DNA containing c-Myc binding sites was assessed via quantitative real-time PCR. The primers used for ChIP are available on request.

### 2.6. Immunocytochemistry

Reprogrammed cells were immunolabeled with anti-mouse Nanog antibodies (Calbiochem, San Diego, CA, USA or Cell Signaling Technology, Danvers, MA, USA) to assess the reprogramming efficiency. A VectaStain ABC kit and ImmPACT DAB substrate (Vector Laboratories, Newark, CA, USA) were used according to the manufacturer’s protocol. Following visualization of Nanog-positive colonies with DAB, the number of Nanog-positive colonies were counted under a stereomicroscope.

### 2.7. In Silico Analysis

The predictable MREs in the 3′ UTR to which miR-17-92 can bind were determined using miRanda, miRDB, and Target Scan. A DAVID GO direct cluster was applied to classify the GO terms of biological processes.

### 2.8. Western Blotting

Immunoblotting was performed based on a standard protocol. Cultured cells were lysed and sonicated. Following protein quantification using a BCA Protein Assay kit (Thermo Scientific), the samples were reduced in loading buffer containing bromophenol blue and 2-mercaptoethanol. SDS-PAGE was performed in a 10% polyacrylamide gel. The primary antibodies used in this study were anti-PTEN antibodies (1:1000 dilution, rabbit polyclonal, Cell Signaling Technology), anti-p21 antibodies (1:1000 dilution, mouse monoclonal, Santa Cruz Biotechnology, Dallas, TX, USA), anti-Histone H3 antibodies (1:1000 dilution, Cell Signaling Technology), and anti-β-actin antibodies (1:5000 dilutions, Sigma-Aldrich). Pierce Western Blotting substrate (Thermo Scientific) was used to visualize the signals, and LAS-4000 (Fuji Film, Tokyo, Japan) was used to detect the signals.

### 2.9. Animals and Ethics

Experiments using mice in this study were approved by the Experimental Animal Committee of Ritsumeikan University. We performed animal care and experimental procedures according to the Animal Welfare Committee guidelines of Ritsumeikan University. Transgenic mice carrying the floxed *Pten* gene (*Pten*^fl/fl^) were purchased from Jackson Laboratory, and the homozygotes were maintained. Pregnant females were sacrificed to collect E13.5–14.5 embryos for MEF primary culture.

### 2.10. Statistics

Statistical analysis was performed using a Student’s *t*-test for two-group comparison and a Tukey–Kramer test for multiple group comparison. All the data are expressed as means ± SD. *p* < 0.05 was considered significant.

## 3. Results

### 3.1. Activation of miR-17-92 Transcription by c-Myc

**Figure 1:**
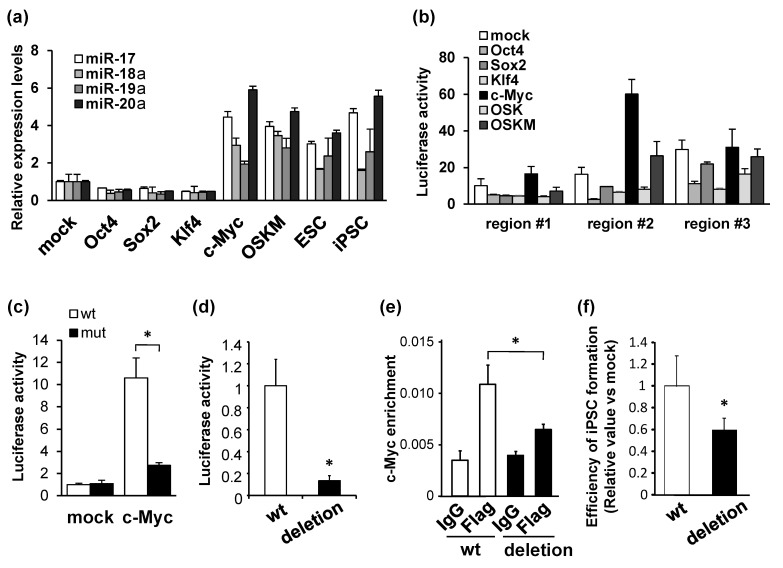
Activation of miR-17-92 transcription by c-Myc. (**a**) Expression levels of miR-17, miR-18a, miR-19a, and miR-20a in MEFs infected with mock or each reprogramming factor indicated. Their levels in mouse pluripotent stem cells (ESCs and iPSCs [15]) are also shown. The expression levels were normalized by values in mock-introduced MEFs. (**b**) Results of luciferase assays using reporters containing one of the three consecutive sections (promoters #1 to #3) in the miR-17-92 promoter region co-transfected with mock, Oct4, Sox2, Klf4, c-Myc, OSK, or OSKM in MEFs. (**c**) Results of the luciferase assay using reporter plasmids containing the wild-type or mutant c-Myc-binding motif of miR-17-92 promoter region #2. The reporter constructs were co-transfected with mock- or c-Myc-expression vectors into HEK 293T cells. (**d**) Results of the luciferase assay using reporter plasmids containing miR-17-92 promoter #2 in HEK 293T cells co-transfected with c-Myc in addition to mock or the CRISPR/Cas9 plasmid targeting the c-Myc-binding motif. (**e**) Quantification of DNA abundance of the miR-17-92 promoter region in wild-type (wt) or c-Myc-binding motif-deleted MEFs, treated with c-Myc-Flag x3. (**f**) Nanog-positive colony numbers of iPSCs reprogrammed from wt or c-Myc-binding motif-deleted MEFs. Data are shown as mean ± SD; * *p* < 0.05 vs. wt.

We examined the levels of miRNAs transcribed from the miR-17-92 cluster during iPSC reprogramming, and attempted to identify the upstream factor(s) involved in miR-17-92 expression. We performed quantitative reverse transcription PCR analysis of MEFs introduced individually or in combination with reprogramming factors (OSKM). OSKM or c-Myc alone markedly elevated the expression levels of miR-17, miR-18a, miR-19a, and miR-20a. These levels were similar to those in undifferentiated ESCs and iPSCs. However, Oct4, Sox2, and Klf4 decreased their expression levels (Figure 1a). Since c-Myc is a reprogramming factor that induces miR-17-92 expression during iPSC reprogramming, we next explored the c-Myc binding sites in the miR-17-92 promoter region. We constructed luciferase reporter plasmids containing one of the three consecutive sections 2.1 kb upstream of the miR-17-92 locus, which are conserved in several mammalian species. Additionally, we transfected each reporter construct into the MEFs infected with Oct4, Sox2, Klf4, c-Myc, OSK, or OSKM. Compared to the mock-transfected cells in each region, higher luciferase activity was induced by c-Myc and OSKM in region #2 containing the c-Myc-binding motif, whereas only minor changes were observed in regions #1 and #3 induced by individual and combined reprogramming factors (Figure 1b). We also found that c-Myc-induced transcriptional activation was reduced in a reporter plasmid with a mutation of the c-Myc-binding site in promoter region #2 (Figure 1c). Additionally, we used the CRISPR/Cas9 system to delete the c-Myc-binding motif in region #2. c-Myc-dependent transcriptional activities were significantly lower in HEK 293T cells transfected with the deletion mutant reporter than in cells transfected with wild-type reporter plasmids (Figure 1d). Then, we established mutant MEFs in which the c-Myc-binding motif was deleted via gene editing. A chromatin immunoprecipitation assay using anti-Flag antibodies revealed that the DNA abundance of miR-17-92 promoter region #2 increased in wild-type MEFs infected with Flag-tagged c-Myc. However, this increase was abolished in the mutant MEFs in which the c-Myc-binding motif was deleted via gene editing (Figure 1e). Furthermore, the efficiency of iPSC generation by OSKM using these mutant MEFs was significantly lower than that using wild-type MEFs (Figure 1f). These results suggest that c-Myc acts as an upstream regulator of miR-17-92 expression, which is involved in iPSC reprogramming by OSKM.

### 3.2. miR-17-92 Is Involved in Mouse iPSC Formation

**Figure 2:**
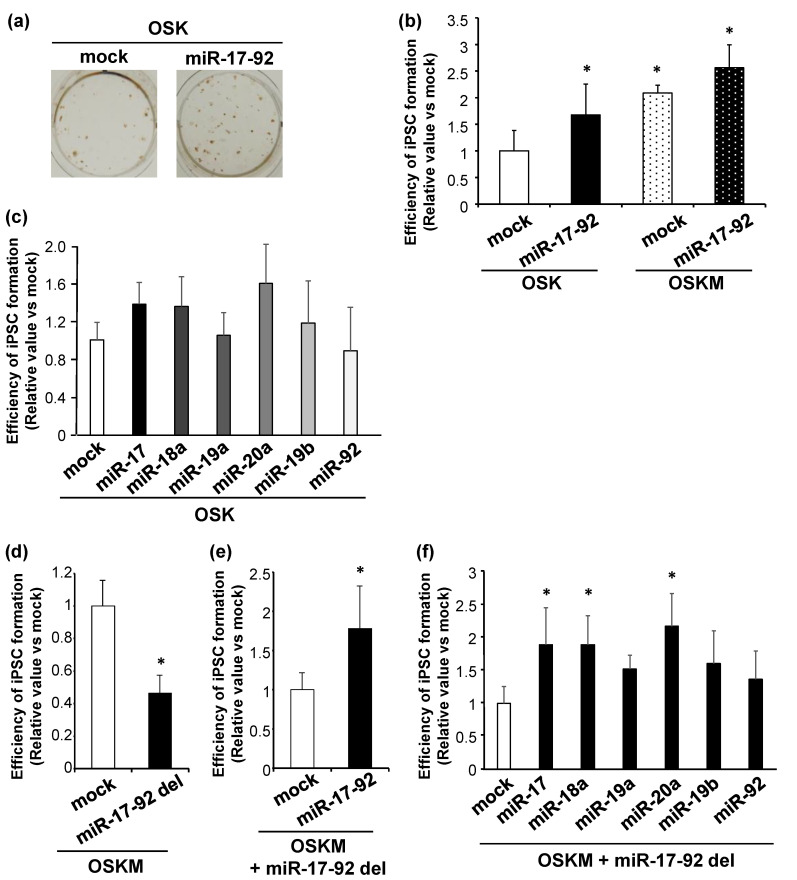
miR-17-92 is involved in efficient iPSC formation. (**a**) Representative images of Nanog-positive iPSC colonies induced by OSK + mock (left) or OSK + miR-17-92 (right). (**b**) Averaged numbers of Nanog-positive iPSC colonies reprogrammed by OSK or OSKM from MEFs co-infected with mock or miR-17-92 lentiviral vectors. * *p* < 0.05 vs. OSK-mock. (**c**) The efficiency of Nanog-positive iPSC colony production by OSK from MEFs co-infected with individual miRNAs composing miR-17-92. (**d**) The production efficiency of Nanog-positive iPSC colonies reprogrammed from OSKM-infected MEFs with or without miR-17-92 deletion. (**e**,**f**) The efficiency of OSKM-induced iPSC production from miR-17-92-deleted MEFs with or without lentiviral overexpression of miR-17-92 (**e**), miR-17, miR-18a, miR-19a, miR-19b, miR-20a, or miR-92 (**f**). Results were normalized by values in mock-infected OSK-reprogrammed cells. * *p* < 0.05 vs. OSKM-mock (**d**–**f**).

Next, we investigated whether miR-17-92 promoted the efficiency of OSK- or OSKM-induced reprogramming. Lentiviral transfection of miR-17-92 was performed in MEFs coinfected with retroviral OSK or OSKM. Immunostaining revealed that miR-17-92 increased the number of Nanog-positive iPSC colonies reprogrammed with OSK or OSKM (Figure 2a,b). This increase was more pronounced in MEFs transfected with OSK (Figure 2b). We then tested which individual miRNAs comprising miR-17-92 were most involved in the enhancement of reprogramming efficiency. We individually overexpressed miR-17, miR-18a, miR-19a, miR-20a, miR-19b, and miR-92 in MEFs co-infected with OSK and found that miR-17, miR-18a, and miR-20a improved the efficiency (Figure 2c). We then tested how much miR-17-92 contributes to a c-Myc-dependent increase in OSKM-reprogramming efficiency, and sought to determine which miRNA derived from the miR-17-92 cluster enhances reprogramming. To address these questions, we used the CRISPR/Cas9 system to delete the miR-17-92 cluster region in MEFs reprogrammed with OSKM. miR-17-92 deficiency resulted in a significant reduction in iPSC colony formation (Figure 2d), which was reversed by the lentiviral overexpression of miR-17-92 (Figure 2e). We also examined whether individual miRNAs derived from the miR-17-92 cluster could rescue the decreased iPSC production caused by miR-17-92 deletion. There was a significant increase in OSKM-induced iPSC colony formation upon the overexpression of individual miRNAs, particularly that of miR-17, miR-18a, and miR-20a. These results demonstrate that miR-17-92 is a potential reprogramming enhancer via miR-17, miR-18a, and miR-20a. Since this effect is more evident in reprogramming induced by OSK than by OSKM, miR-17-92 may partially share c-Myc-dependent mechanisms associated with efficient iPSC production.

### 3.3. Potential Targets of Individual miRNAs Comprising miR-17-92

**Figure 3:**
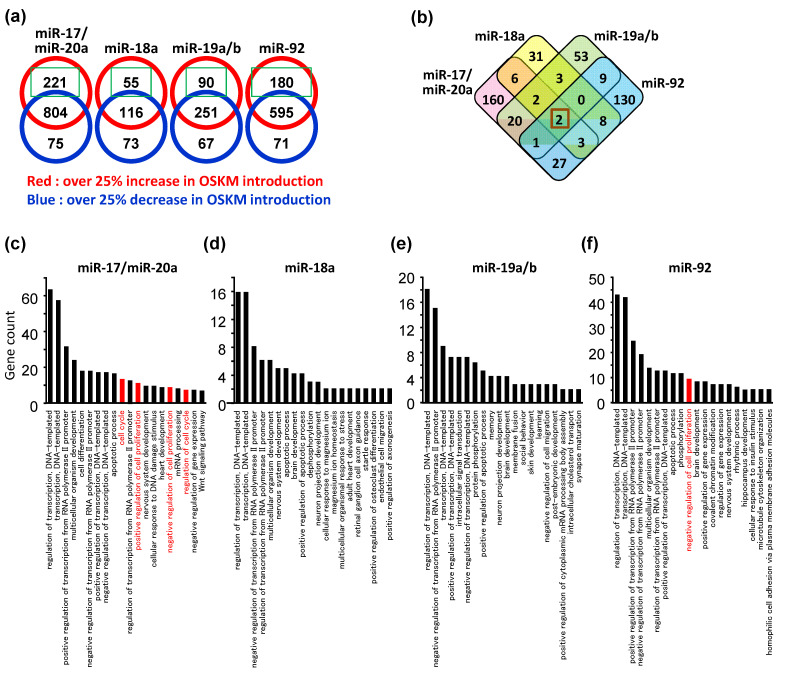
Potential targets of individual miRNAs comprising miR-17-92. (**a**) Numbers of predicted target genes for each individual miRNA comprising the miR-17-92 cluster with over 1.25-fold changes in OSKM-introduced MEFs. (**b**) Venn diagram showing the numbers of overlapped miR-17-92 target genes more than 25% upregulated by OSKM. (**c**–**f**) GO terms enriched with OSKM-upregulated genes with binding motifs of miR-17/20a (**c**), miR-18a (**d**), miR-19a/b (**e**), or miR-92a (**f**).

We further investigated corresponding target genes for miR-17-92 expression. The genes upregulated by OSKM introduction included miR-17-92 target genes responsible for iPSC formation. Thus, we performed an in silico search for the target genes of the miR-17-92 cluster using microarray data. For this analysis, since miR-17 and miR-20a share the same binding motif, we categorized miR-17-92 into miR-17/20a, miR-18a, miR-19a/b, and miR-92a. TargetScan identified 221, 55, 90 and 180 genes with a binding motif for miR-17/20a, miR-18a, miR-19a/b, and miR-92a, respectively, from the genes with over a 25% increase in OSKM-infected MEFs. (Figure 3a). The resultant Venn diagram indicates the number of overlapping target genes from among the miRNAs (Figure 3b). We identified two candidate genes, phosphatase and tensin homologue (*Pten*) and *D1Ertd622e*, which were potential targets shared by all the miRNAs derived from the miR-17-92 cluster. Furthermore, gene ontology (GO) term analysis revealed that the top 20 gene categories altered by OSKM included cell cycle-related categories among the target genes of miR-17/20a and miR-92 (Figure 3c–f, Tables S1–S4). *Pten*, a well-known tumor repressor, was the only gene that had a common target motif with miR-17/20a, miR-18a, miR-19a/b, and miR-92a (Table S5) from among the individual genes categorized in the cell cycle-related GO terms. In addition, cyclin-dependent kinase inhibitor 1 (*Cdkn1a*), known as *p21*, a negative regulator of reprogramming to pluripotency, together with *p53* [15,18], was included among the miR-17/20a target genes (Table S5).

### 3.4. PTEN and p21 Are Downregulated by miR-17 and miR-20a

**Figure 4:**
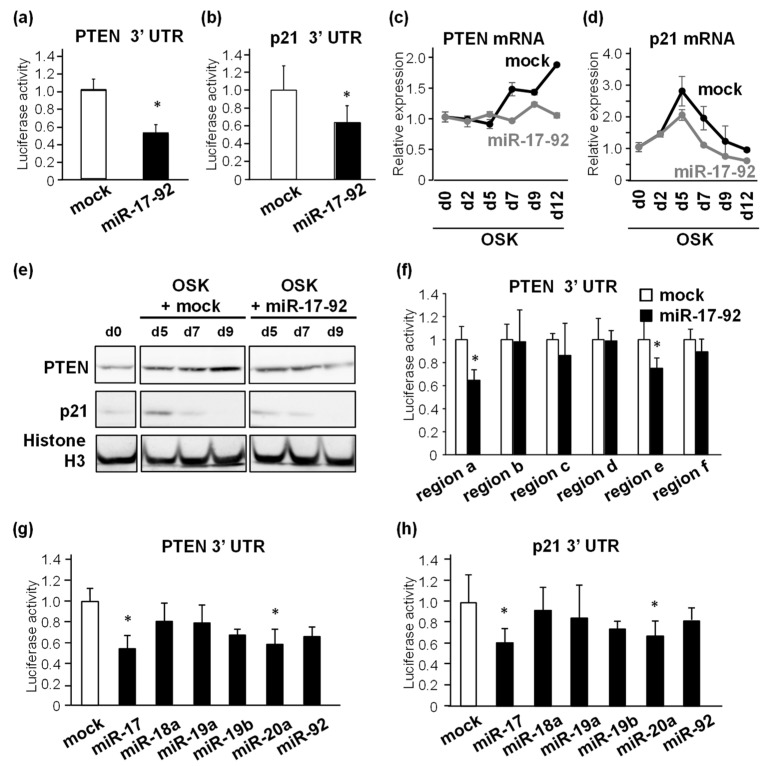
PTEN and p21 are downregulated by miR-17 and miR-20a. (**a**,**b**) Luciferase activities of the 3′ UTR reporter of PTEN (**a**) or p21 (**b**) mRNA in HEK 293T cells with or without miR-17-92 overexpression. * *p* < 0.05 vs. mock. (**c**,**d**) Dynamic changes in the mRNA expression levels of PTEN (**c**) and p21 (**d**) during the reprogramming process in OSK-introduced MEFs co-infected with mock or miR-17-92. (**e**) Protein levels of PTEN and p21 on indicated days from OSK-introduced MEFs co-infected with mock or miR-17-92. Histone H3 was used as a loading control. (**f**) Luciferase activities of the six consecutive regions of the 3′ UTR reporter of the PTEN mRNA (regions **a**–**f**) in HEK 293T cells with or without miR-17-92 overexpression. (**g**,**h**) Luciferase activities of 3′ UTR of PTEN (**g**) or p21 (**h**) mRNA in HEK 293T cells transfected with mock, miR-17, miR-18a, miR-19a, miR-19b, miR-20a, or miR-92. * *p* < 0.05 vs. mock.

The inhibition of PTEN improves iPSC production, and the inhibition of the tumor-suppressing p53/p21 pathway results in increased efficiency of pluripotency, as reported by us and others [15,18,19]. Therefore, we investigated whether PTEN and p21 were involved in miR-17-92-mediated iPSC production. The direct regulation of PTEN and p21 expression by miR-17-92 was analyzed using luciferase reporter assays. We constructed luciferase reporter plasmids containing the 3′ UTR of PTEN or p21 mRNA and transfected them into HEK 293T cells, which exhibit no expression of mouse miRNAs, together with mock- or miR-17-92-expression plasmids. Luciferase activity was significantly decreased in the presence of miR-17-92, suggesting that miR-17-92 suppressed PTEN and p21 expression post-transcriptionally (Figure 4a,b). The dynamic changes in PTEN and p21 mRNA levels during iPSC reprogramming were assessed. PTEN expression increased incrementally from the mid-to-late phases of reprogramming, and its level was highest on day 12 of OSK transduction (Figure 4c). p21 expression was also elevated by OSK introduction, reaching its peak level on day 5, and subsequently decreasing to the basal level (Figure 4d). However, in the presence of miR-17-92, the upregulation of PTEN in the late phase was abolished (Figure 4c), and the p21 peak level was also obviously reduced in the early- to mid-phase (Figure 4d). Similar results were observed for the protein levels, although the decrease in p21 level was modest (Figure 4e). These results suggest that miR-17-92 post-transcriptionally downregulates PTEN and p21 expression in a stage-specific manner during iPSC reprogramming.

To identify the binding sites of miR-17-92 in the PTEN 3′ UTR, which is approximately 6 kb long, we constructed luciferase reporter plasmids containing each of its six consecutive regions (a–f) and transfected them into HEK 293T cells with or without the miR-17-92 expression plasmid. miR-17-92 suppressed the luciferase activity in regions a, c, and e (Figure 4f). Database analysis predicted that miR-17 and miR-20a could bind to the a and e regions from among the miR-17-92 cluster-comprising miRNAs. A reporter assay was performed to examine whether miR-17 and miR-20a reduced the mRNA levels of PTEN, and p21. HEK 293T cells were infected with vectors encoding individual miRNAs of the miR-17-92 cluster, and then, transfected with the PTEN or p21 3′ UTR reporter plasmids. We found that the luciferase activity of the PTEN 3′ UTR reporter was significantly suppressed by miR-17-92, miR-17, and miR-20a (Figure 4g). p21 3′ UTR reporter activity was also decreased by miR-17-92, miR-17, and miR-20a (Figure 4h). Taken together, miR-17 and miR-20a may correspond with the miR-17-92-mediated downregulation of PTEN and p21, thereby enhancing iPSC production.

### 3.5. iPSC Production Is Downregulated by PTEN ceRNA against miR-17-92

**Figure 5:**
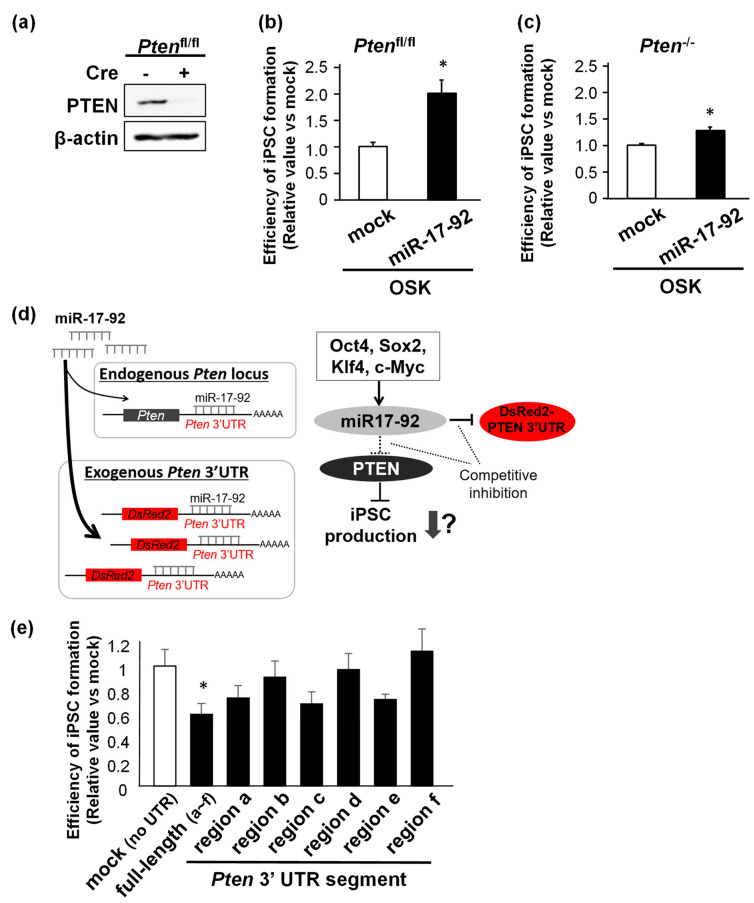
iPSC production downregulated by PTEN ceRNA against miR-17-92. (**a**) Western blotting of PTEN in *Pten*^fl/fl^ MEFs with or without Cre recombinase transduction. (**b**,**c**) Averaged numbers of Nanog-positive iPSC colonies induced from *Pten*^fl/fl^ (**b**) and *Pten*^−/−^ (**c**) MEFs transduced with OSK with or without miR-17-92 overexpression. (**d**) A schematic illustration of the experiment to test the effect of PTEN 3′ UTR segments as “miRNA sponges” on iPSC production. (**e**) The production efficiency of Nanog-positive iPSC colonies reprogrammed from OSKM-introduced MEFs co-infected with mock (no UTR) or DsRed fused to each region (a to f) of the PTEN 3′ UTR. Each segment of the PTEN 3′ UTR contains various numbers of binding motifs to miRNAs derived from the miR-17-92 cluster. Results were normalized by values in mock-infected OSK-reprogrammed cells. * *p* < 0.05 vs. mock.

Next, we examined how PTEN affected the miR-17-92-mediated enhancement of mouse iPSC production. We used MEFs carrying a floxed *Pten* gene (*Pten*^fl/fl^). Transduction with Cre recombinase converted the floxed allele into the knockout *Pten* gene and almost completely repressed PTEN protein expression in these MEFs (Figure 5a). The overexpression of miR-17-92 increased iPSC production efficiency in *Pten*^−/−^ MEFs to a lesser extent than in *Pten*^fl/fl^ MEFs (Figure 5b,c). This suggests that PTEN is not the only miR-17-92-target affecting iPSC reprogramming efficiency. p21 is another target of miR-17-92 that inhibits iPSC reprogramming. We hypothesized that PTEN mRNA and other miR-17-92 targets, including p21 mRNA, may act as competing endogenous RNAs (ceRNAs), which are RNAs sharing miRNA recognition elements (MREs) with other RNAs (such as mRNA and long non-coding RNA), as common targets against the miRNA [20]. Depending on the miRNA affinity, variable post-transcriptional regulation of one or more genes may occur among all miRNA targets [20,21]. To examine the above hypothesis, we constructed lentiviral vectors expressing a DsRed coding region fused with each segment of the six consecutive regions (a–f) or the full-length PTEN 3′ UTR. Each of these regions contained various numbers of binding motifs for individual miRNAs comprising miR-17-92, such that the exogenous expression of these segments allowed for the competitive absorption of endogenous miRNAs that bind to the same motifs (Figure 5d). Accordingly, we overexpressed the full-length or a single segment of the PTEN 3′ UTR in MEFs co-infected with OSKM to examine the effects of these “miRNA sponges” on iPSC reprogramming efficiency. Maximum reduction in the Nanog-positive iPSC colony number was observed in cells treated with the full-length PTEN 3′ UTR (Figure 5e). iPSC production was also decreased in cells expressing regions a and e, to which miR-17 and miR-20a can bind (Figure 5e). Since both the PTEN 3′ UTR and p21 3′ UTR contain binding motifs for miR-17 and miR-20a, we speculated that these miRNAs were responsible for the miR-17-92-dependent enhancement of iPSC formation through PTEN/p21 inhibition.

### 3.6. Post-Transcriptional Regulation of PTEN and p21 by miR-17-92 in the Context of ceRNA

**Figure 6:**
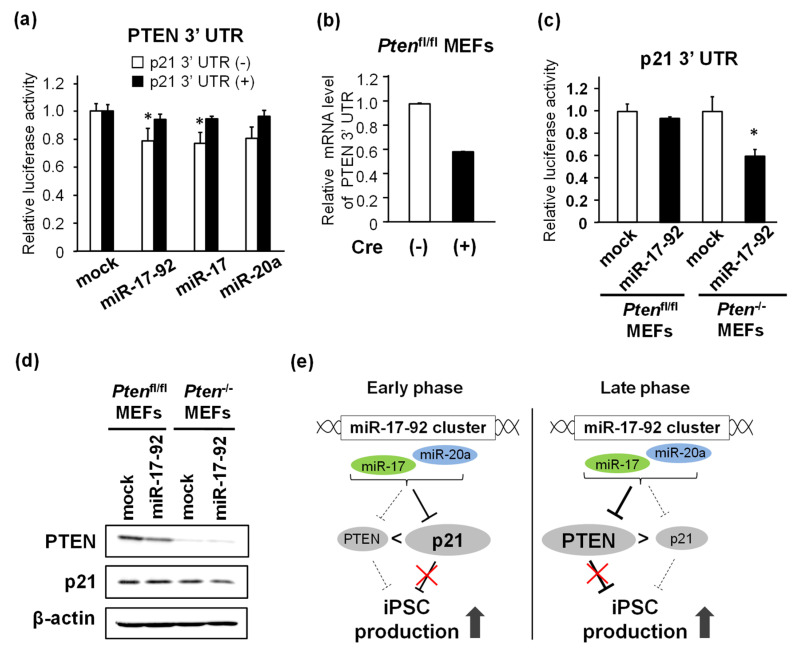
Post-transcriptional regulation of PTEN and p21 by miR-17-92 in the context of ceRNA. (**a**) Luciferase activity of the PTEN 3′ UTR reporter in HEK 293T cells transfected with mock, miR-17-92, miR-17, or miR-20a in the presence or absence of the p21 3′ UTR. (**b**) The knockout efficiency of the PTEN 3′ UTR mRNA level in *Pten*^fl/fl^ MEFs with or without Cre transduction. (**c**) Luciferase activity of the p21 3′ UTR reporter in *Pten*^fl/fl^ and *Pten*^−/−^ MEFs treated with miR-17-92. (**d**) The protein levels of PTEN and p21 in *Pten*^fl/fl^ and *Pten*^−/−^ MEFs treated with mock or miR-17-92. β-action was used as a loading control. (**e**) Working hypothesis of post-transcriptional regulation of PTEN and p21 by miR-17-92 in the context of ceRNA. * *p* < 0.05 vs. mock.

To examine the effect of p21 3′ UTR on miR-17-92-mediated PTEN downregulation, luciferase assays were performed using 293T cells, in which endogenous p21 is poorly expressed. These cells were transfected with mock, miR-17-92, miR-17, or miR-20a, together with mock (no UTR) or DsRed/p21 3′ UTR fusion mRNA. The exogenous expression of miR-17-92, miR-17, and miR-20a significantly reduced the luciferase activity of the PTEN 3′ UTR reporter; however, this reduction was almost abolished by co-transfection with the DsRed/p21 3′ UTR (Figure 6a). Next, we tested whether miR17-92-dependent p21 suppression was affected by the endogenous PTEN 3′ UTR. Using *Pten*^fl/fl^ MEFs with or without Cre recombinase transduction, we performed the PTEN 3′ UTR luciferase experiments. In *Pten*^fl/fl^ MEFs transduced with Cre recombinase, endogenous PTEN 3′ UTR expression significantly reduced (Figure 6b). In *Pten*^fl/fl^ MEFs, the overexpression of miR-17-92 only resulted in a minor decrease in p21 3′ UTR reporter activity. However, in Cre recombinase-transduced cells (*Pten*^−/−^ MEFs), the endogenous PTEN 3′ UTR level was reduced, and luciferase activity was significantly decreased by miR-17-92 (Figure 6c). Western blotting revealed that miR-17-92 decreased PTEN levels in *Pten*^fl/fl^ MEFs and that p21 protein levels were repressed only in *Pten*^−/−^ MEFs (Figure 6d). These results suggest that PTEN mRNA and p21 mRNA act as ceRNAs and are competitively downregulated by miR-17 and miR-20a, which are transcribed from the miR-17-92 cluster.

## 4. Discussion

In the present study, we investigated the role of the miR-17-92 cluster in mouse iPSC production. First, we found that the reprogramming factor c-Myc increased the expression levels of miRNAs in the miR-17-92 cluster. We identified a c-Myc-binding site in the proximal enhancer region of the miR-17-92 cluster that may be involved in iPSC production. Second, miR-17-92 overexpression increased the number of iPSC colonies, whereas CRISPR/Cas9-based deletion of the miR-17-92 cluster reduced iPSC reprogramming efficiency. Notably, among the miRNAs transcribed from the miR-17-92 cluster, the overexpression of either miR-17 or miR-20a significantly recovered the efficiency that was decreased by miR-17-92 deletion. Third, we found that PTEN and p21, the putative target genes of miR-17 and miR-20a, respectively, were competitively downregulated by miR-17-92. Notably, the reduced expression levels of p21 in the early phase and PTEN in the mid-to-late phase of the reprogramming process may be regulated by miR-17-92 in a ceRNA manner.

We and others have previously demonstrated that p53 deficiency dramatically increases reprogramming efficiency through the inhibition of apoptosis and p21-dependent cell cycle arrest; moreover, p21, a target gene of tumor suppressor p53, significantly reduces iPSC production [15,18,22,23,24]. PTEN is also a tumor suppressor that stabilizes p53 through inactivation of the E3 ubiquitin ligases MDM2 and MDMX [25]. Therefore, targeting p21 and PTEN increased iPSC production. The present study demonstrated that miR-17-92 contributed towards enhancing iPSC reprogramming efficiency through the phase-dependent downregulation of PTEN and p21 expressions (Figure 6e). During the reprogramming process, miR-17-92 post-transcriptionally repressed the expression of p21 and PTEN in the early and mid-to-late phases of reprogramming, respectively. The inhibitory effect of miR-17-92 on PTEN appeared to be weak in the early-to-mid phase. This weak inhibition may be attributed to ceRNA-mediated regulation. In general, miRNAs inhibited the stability and translation of mRNAs by forming an RNA-induced silencing complex that bound to a partially complementary sequence called MRE in their target genes. ceRNAs are a variety of RNA, including long non-coding RNA and mRNA with the same MRE, and they competitively share the same miRNA pool [20,21]. During iPSC reprogramming, we demonstrated that the miR-17-92-dependent downregulation of PTEN was influenced by p21 transcript levels, suggesting that miR-17-92 preferentially repressed p21 rather than PTEN in the early-to-mid phase when p21 mRNA expression was upregulated. In human cells, ceRNA against *PTEN* is shared with a pseudogene, *PTENP1*, which is encoded by a similar *PTEN* locus, producing an inactive form of the PTEN transcript [26,27]. Although *PTENP1* is not conserved in the mouse genome, ZEB2 mRNA has been reported to be a PTEN ceRNA in mouse cells [28]. ZEB2 mRNAs may sponge miRNAs and perturb the repression of other target genes, causing the downregulation of PTEN at the protein level. The attenuation of ZEB2 commonly results in activation of the PI3K/Akt pathway, a downstream target of PTEN, thereby enhancing cell transformation in human melanoma [28]. Although many other target genes may also share miR-17-92 with other ceRNAs, our data demonstrated that PTEN expression was competitively regulated by p21 mRNA, acting as a sponge for miR-17-92 during iPSC reprogramming. Furthermore, *Pten*^−/−^ MEFs displayed a significant decrease both in p21 3′ UTR luciferase activity and the p21 protein level through the overexpression of miR-17-92 (Figure 5c,d). Thus, the miR-17-92-mediated downregulation of p21 may be weakened in the late phase of reprogramming associated with the upregulation of PTEN.

In conclusion, we showed that c-Myc contributes to increased miR-17-92 expression and iPSC production efficiency during OSKM-based reprogramming. Moreover, miR-17-92, particularly miR-17 and miR-20a, may phase-dependently repress the expression of p21 and PTEN via ceRNA, thereby improving the efficiency of reprogramming to pluripotency. Although further studies are needed to compare the effectiveness of other miRNAs, including the miR-290 cluster, miR-106b, miR-302, and miR-372, as reprogramming enhancers [5,6,11], our findings provide insights into the molecular mechanisms underlying iPSC production via ceRNAs.

## Data Availability

The datasets generated and analyzed during the present study are available from the corresponding author on reasonable request.

## Supplementary Materials

The following supporting information can be downloaded at: https://www.mdpi.com/article/10.3390/biomedicines11061737/s1, Table S1: GO terms enriched with OSKM-upregulated genes with miR-17/miR-20a-binding motifs; Table S2: GO terms enriched with OSKM-upregulated genes with miR-18a-binding motifs; Table S3: GO terms enriched with OSKM-upregulated genes with miR-19a/b-binding motifs; Table S4: GO terms enriched with OSKM-upregulated genes with miR-92a-binding motifs; Table S5: OSKM-upregulated genes enriched in GO term “regulation of cell cycle”.
