# Supplementary material for: c-Myc/microRNA-17-92 Axis Phase-Dependently Regulates PTEN and p21 Expression via ceRNA during Reprogramming to Mouse Pluripotent Stem Cells
Source: Biomedicines. 2023 Jun 16;11(6):1737. doi: 10.3390/biomedicines11061737 (PMC10296502; doi:10.3390/biomedicines11061737)
Supplement: Supplementary file 1 [file biomedicines-11-01737-s001.zip › biomedicines-2417962-supplementary.pdf]

Supplementary Materials

Table S1. GO terms enriched with OSKM-upregulated genes with miR-17/miR-20a-binding motifs.

GO Terms	Gene Counts
Regulation of transcription, DNA-templated	63
Transcription, DNA-templated	57
Positive regulation of transcription fro M rna polymerase ii promoter	31
Multicellular organism development	24
Negative regulation of transcription from RNA polymerase II promoter	18
Cell differentiation	18
Positive regulation of transcription, DNA-templated	17
Negative regulation of transcription, DNA-templated	17
Apoptotic process	16
Cell cycle	13
Regulation of transcription from RNA polymerase II promoter	12
Positive regulation of cell proliferation	11
Nervous system development	10
Cellular response to DNA damage stimulus	10
Heart development	9
Negative regulation of cell proliferation	9
Mrna processing	8
Regulation of cell cycle	7
Negative regulation of gene expression	7
Wnt signaling pathway	6

Table S2. GO terms enriched with OSKM-upregulated genes with miR-18a-binding motifs.

GO Terms	Gene Counts
Transcription, DNA-templated	16
Regulation of transcription, DNA-templated	16
Negative regulation of transcription from RNA polymerase II promoter	8
Regulation of transcription from RNA polymerase II promoter	6
Multicellular organism development	6
Nervous system development	5
Apoptotic process	5
Brain development	4
Positive regulation of apoptotic process	4
Dephosphorylation	3
Neuron projection development	3
Cellular response to magnesium ion	2
Magnesium ion homeostasis	2
Multicellular organismal response to stress	2
Adult heart development	2
Retinal ganglion cell axon guidance	2
Startle response	2
Positive regulation of osteoclast differentiation	2
Endothelial cell migration	2
Positive regulation of axonogenesis	2

Table S3. GO terms enriched with OSKM-upregulated genes with miR-19a/b-binding motifs.

GO Terms	Gene Counts
Regulation of transcription, DNA-templated	18
Transcription, DNA-templated	15
Negative regulation of transcription from RNA polymerase II promoter	9
Intracellular signal transduction	7
Positive regulation of transcription, DNA-templated	7
Negative regulation of transcription, DNA-templated	7
Protein phosphorylation	6
Positive regulation of apoptotic process	5
Memory	4
Neuron projection development	4
Brain development	4
Membrane fusion	3
Social behavior	3
Skin development	3
Learning	3
Negative regulation of cell migration	3
Post-embryonic development	3
Positive regulation of cytoplasmic mrna processing body assembly	2
Intracellular cholesterol transport	2
Synapse maturation	2

Table S4. GO terms enriched with OSKM-upregulated genes with miR-92a-binding motifs.

GO Terms	Gene Counts
Regulation of transcription, DNA-templated	43
Transcription, DNA-templated	42
Positive regulation of transcription from RNA polymerase II promoter	24
Negative regulation of transcription from RNA polymerase II promoter	19
Multicellular organism development	14
Regulation of transcription from RNA polymerase II promoter	13
Positive regulation of transcription, DNA-templated	13
Apoptotic process	11
Phosphorylation	11
Negative regulation of cell proliferation	9
Brain development	8
Positive regulation of gene expression	8
Covalent chromatin modification	7
Regulation of gene expression	7
Nervous system development	7
Rhythmic process	6
Hippocampus development	5
Cellular response to insulin stimulus	5
Microtubule cytoskeleton organization	5
Homophilic cell adhesion via plasma membrane adhesion molecules	5

Table S5. OSKM-upregulated genes enriched in GO term "regulation of cell cycle".

miRNAs	Target Genes
miR-17/20a	Arid4b, Bid, Ccnd1, Cdkn1a, Clock, E2f2, Fzd3, Kat2b, Kif13a, Kif23, Mecp2, Myo19, Myo9a, Plcb1, Pten, Ptpn3, Pura, Rb1, Rbl2, Rpa2, Tal1, Trim37, Trp53inp1, Ubxn2b
miR-18a/b	Fam83d, Hecw2, Hnrnpu, L3mbtl, Mecp2, Plcb1, Pten, Trp53inp1, Ubxn2b
miR-19	Jmy, Mecp2, Notch2, Pten, Zfp207
miR-92a	Btg2, Cdk6, Ddx3x, Fbxw7, Hnrnpu, Jmy, Kat2b, Klf4, Mdm4, Pten, Rad21, Rbl2, Stag2
